# Mutation in *phcA* Enhanced the Adaptation of *Ralstonia solanacearum* to Long-Term Acid Stress

**DOI:** 10.3389/fmicb.2022.829719

**Published:** 2022-05-26

**Authors:** Ying Liu, Xi Tan, Yanxin Pan, Jiamin Yu, Yiran Du, Xiaojiao Liu, Wei Ding

**Affiliations:** ^1^College of Plant Protection, Southwest University, Chongqing, China; ^2^Sichuan Company of China National Tobacco Corporation, Chengdu, China

**Keywords:** *Ralstonia solanacearum*, experimental evolution, long-term acid stress, acid adaptation, *phcA* gene

## Abstract

Bacterial wilt, caused by the plant pathogen *Ralstonia solanacearum*, occurs more severely in acidified soil according to previous reports. However, *R. solanacearum* cannot grow well in acidic environments under barren nutrient culture conditions, especially when the pH is lower than 5. With the worsening acidification of farmland, further determination of how *R. solanacearum* adapts to the long-term acidic environment is worthwhile. In this study, experimental evolution was applied to evaluate the adaptability and mechanism of the *R. solanacearum* experimental population responding to long-term acid stress. We chose the CQPS-1 strain as the ancestor, and minimal medium (MM medium) with different pH values as the culture environment to simulate poor soil. After 1500 generations of serial passage experiments in pH 4.9 MM, acid-adapted experimental strains (denoted as C49 strains) were obtained, showing significantly higher growth rates than the growth rates of control experimental strains (serial passage experiment in pH 6.5 MM, denoted as C65 strains). Competition experiments showed that the competitive indices (CIs) of all selected clones from C49 strains were superior to the ancestor in acidic environment competitiveness. Based on the genome variation analysis and functional verification, we confirmed that loss of function in the *phcA* gene was associated with the acid fitness gain of *R. solanacearum*, which meant that the inactivation of the PhcA regulator caused by gene mutation mediated the population expansion of *R. solanacearum* when growing in an acidic stress environment. Moreover, the swimming motility of acid evolution strains and the *phcA* deletion mutant was significantly enhanced compared to CQPS-1. This work provided evidence for understanding the adaptive strategy of *R. solanacearum* to the long-term acidic environment.

## Introduction

*Ralstonia solanacearum*, one of the top ten plant pathogenic bacteria in the world, is considered a variable compound species due to its large host range, wide distribution, and rich genetic diversity (Genin and Denny, 2012; Mansfield et al., 2012). Wide geographic distribution and host range make *R. solanacearum* face and adapt to various environments, such as poor nutrient environments and overly acidic environments. The soil environment is an important living place for *R. solanacearum*, especially in the fallow period. According to reports, *R. solanacearum* has a strong ability to survive in the soil (Graham and Lloyd, 1979).

Soil acidification is one of the most serious soil problems in the current ecological environment and agricultural production process (Guo et al., 2010; Kochian et al., 2015). Acidification not only affects plant growth and nutrient absorption but is also closely related to the occurrence of diseases. Previous studies have shown that soil acidification is related to the occurrence of bacterial wilt and the pathogenicity of *R. solanacearum*: lower pH in acidified soil results in more severe bacterial wilt, and the pathogenic genes of *R. solanacearum* are induced (Li et al., 2017). However, our previous research shows that the growth of *R. solanacearum* is inhibited when the pH is lower than 5.0 in culture with poor nutrient. With the continuous input of human activities such as nitrogen fertilizer, the problem of soil acidification has become increasingly serious (Zhalnina et al., 2015). The effect of a long-term acidic environment on *R. solanacearum* is worthy of attention but has not been reported thus far.

Evolutionary biologists have more specifically studied the limitations and potential of species to adapt to the environment, including abiotic stresses (such as salt stress, heavy metal stress, heat stress, pH stress, and drought or water stress), as well as when the environment changes how species react (Bijlsma and Loeschcke, 2005; Bridle and Vines, 2007; Flowers et al., 2010; Kooyers, 2015). Organisms can respond to these abiotic stresses in many ways. In the face of stress, organisms can adapt through phenotypic plasticity on the one hand, and change their phenotype to adapt to the abiotic environment; on the other hand, organisms can also respond to changes in genotypes that match abiotic conditions through evolutionary adaptation (Westeberhard, 1989; Kawecki and Ebert, 2004). When populations cannot quickly adapt or migrate (dispersion), these populations may be forced to extinction locally, and the remaining part of the surviving population will adapt to the environment and “evolve”. And thus, “experimental evolution and resequence” has a great potential to predict how natural populations will evolve and adapt to major changes in environmental conditions (Phillips and Burke, 2021). Genomes are a very useful resource to understand the evolutionary process and adaptability of the species. *R. solanacearum* strain GMI1000 was the first strain subject to whole genome analysis, after which a large number of strain genomes were published (Salanoubat et al., 2002; Peeters et al., 2013). A large amount of genomic information provides a basis for analyzing this population. More importantly, the extremely large possibility of variation within the genomes of the *R. solanacearum* species complex determines its strong adaptability and polymorphism (Salanoubat et al., 2002; Genin and Boucher, 2004). However, there is currently no information on the genomes of *R. solanacearum* strains adapted to acidic soil environments.

To explore how *R. solanacearum* survives and adapts to a low pH environment for a long time, the methods of experimental evolution and genome resequencing were selected for evaluation in this study, aiming to clarify the molecular mechanism of the *R. solanacearum* response to acid stress. The results showed that loss of function in the *phcA* gene was associated with the gain in acid fitness of *R. solanacearum*, which laid the foundation for theoretical research on the adaptive evolution of the *R. solanacearum* strain.

## Materials and Methods

### Minimal Medium With Different pH Values as the Acidic and Control Environment

This study simulated poor soil with minimal medium (MM medium). The MM medium is equivalent to 1/4 M63 medium (Boucher et al., 1985). The basic composition of MM medium contained FeSO_4_⋅7H_2_O, (NH_4_)_2_SO_4_, MgSO_4_, and KH_2_PO_4_. When cultivating *R. solanacearum*, glucose was used as the carbon source, supplemented at a final concentration of 20 mM. The different pH values of MM medium were adjusted by KOH. The pH of the MM medium was adjusted to 6.5 with KOH as the control environment and without KOH to adjust the pH to an acidic environment (pH 4.9) for the experiment.

### Bacterial Strains and Culture Conditions

*Ralstonia solanacearum* strain CQPS-1 (phylotype I/biovar 3) with known genome information was used as the original strain in this study (Liu et al., 2017). *R. solanacearum* strain CQPS-1 was grown at 30°C in BG medium, B liquid medium, or MM medium with different pH values based on needs. Growth was detected by measuring the optical density (OD) at 600 nm. The bacterial growth curve was monitored according to Yang et al. (2016). Morphologies of colonies were observed on BG medium supplemented with triphenyltetrazolium chloride (TTC, 0.05 g⋅L^–1^).

*Escherichia coli* strains were grown at 37°C in Luria-Bertani (LB) medium. When necessary, antibiotics were added to the media at the following final concentrations: ampicillin (Amp, 100 mg⋅L^–1^), kanamycin (km, 50 mg⋅L^–1^), polymyxin B (PB, 50 mg⋅L^–1^), gentamycin (Gm, 20 mg⋅L^–1^).

### Long-Term Experimental Evolution of *Ralstonia solanacearum* in an Acidic Environment

**FIGURE 1:**
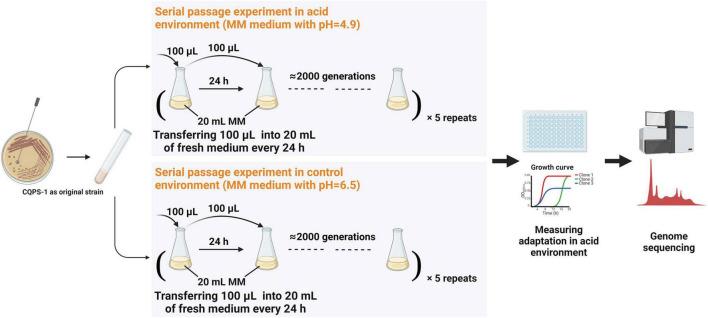
Long-term experimental evolution of CQPS-1 in an acidic environment. MM medium at pH 4.9 was used as the acidic environment, and MM medium (pH 6.5) was used as the control. Cultures were propagated daily by transferring 100 μL of each culture into 20 mL of fresh medium. The figure was created with BioRender.com.

A serial passage experiment was performed on a clone of *R. solanacearum* CQPS-1 using MM medium with different pH values adjusted by KOH. At each SPE (serial passage experiment), bacteria were grown in 20 ml medium for 24 h at 30° with 180 r/min shaking, and then 100 μl of culture was transferred into 20 ml fresh medium (as shown in Figure 1). Each treatment had five independent cultures (equivalent to five parallel replicates). The number of bacterial generations at each SPE was estimated by comparing the number of cells at SPE_*n*_ and the effective number of cells at SPE_*n+*1_, which was just an estimate, and the actual population generation should be larger than the estimated value population size. Finally, glycerol stock of strains stored at −80°C at point time, which can be recovered for relevant evaluation.

### Monitoring *in vitro* Growth Rates of Evolved Populations

The growth rates of evolved populations growing in an acidic environment were measured in MM cultures at pH 4.9. One clone was randomly isolated from every five independent populations generated by the serial passage experiment. Overnight-cultured bacterial suspension (1 μl) was inoculated in 199 μl MM medium (pH 4.9) in 96-well plates. Bacterial growth was monitored using a Multiskan GO microplate reader (Thermo Fisher Scientific). Measures of OD_600 *nm*_ were performed every 5 min. The growth rates of each sample within 32 h were determined using the kinetic analysis module of Multiskan GO software. All samples were analyzed in triplicate and calculated to obtain an averaged value.

### Bacterial Competition Assays in the Acidic Environment

The method used to estimate the fitness of *R. solanacearum* clones in acidic environments is based on competition assays with some modifications (Macho et al., 2010). Briefly, the acidic environment fitness of each evolved clone was measured by co-culturing with the ancestral clone in MM medium at pH 4.9. The distinction between experimental clones and original clones is based mainly on colony morphology: all the colonies after the serial passage experiment were small, dark red, with poor fluidity, while the colonies of the original strain CQPS-1 were cream-colored, irregularly shaped, and highly fluid (as shown in Supplementary Figure 1). A 10^8^ colony forming unit (CFU)/ml bacterial suspension containing equal CFU of the experimental clone and the ancestral clone was inoculated in MM (pH 4.9) and grown at 30°C with 180 rpm shaking. After 24 h, serial dilutions of the inoculum were plated onto BG medium, to determine the bacterial ratio between the experimental and ancestral clones. The competitive index (CI) was defined as the experimental/ancestral-clone ratio divided by the ratio in the corresponding inoculum (Baumler et al., 1997; Guidot et al., 2014).

### Genome Resequencing and Detection of Mutations

All genomes of strains were sequenced using Illumina technology. Libraries were constructed and sequenced by Biomarker Technologies Corporation using Illumina’s HiSeq-4000 with 2 × 150-bp paired-end reads, according to the Illumina company protocol. The quality of the raw reads was evaluated and filtered to obtain clean reads for subsequent bioinformatics analysis. Sequence data were mapped to the genome of the original strain CQPS-1 (accession numbers CP016914 and CP016915) (Liu et al., 2017). bwa software is used mainly for the comparison of the obtained short sequences with reference genomes (Li and Durbin, 2009). Picard^1^ was used to filter redundant reads. The HaplotypeCaller algorithm of GATK software and SnpEff have been used to detect and annotate SNPs and small InDels (Mckenna et al., 2010): gVCF was generated for each sample first, then population joint-genotype was performed; finally, the final set of variant sites was obtained after filtering. The filter parameters adopt the default values officially designated by GATK. All filtered polymorphisms were checked by PCR amplification and sequencing with Sanger technology using the primers reported in Supplementary Table 1.

### Construction of Deletion Mutants and Complementation Analyses

Mutants with gene deletions were generated based on homologous recombination by pK18mobsacB as described by Zhang et al. (2015, 2018). Briefly, two flanking fragments of the target gene were amplified from the genomic DNA of CQPS-1. Then, a second round of polymerase chain reaction (PCR) was performed to generate the DNA fragment without the open-reading frame of the target gene. The generated DNA fragment was cloned into pK18mobsacB to obtain the target pK18 plasmid. After the sequence was validated, the target plasmid was delivered from *E. coli* strain S17-1 into *R. solanacearum* strain CQPS-1 by conjugative transfer. A deletion mutant was generated through consecutive homologous recombination events. The successful mutant was verified by PCR. The complementation analysis was performed with a pUC18-mini-Tn7T-Gm-based site-specific chromosomal integration system as previously described (Zhang et al., 2018; Liu et al., 2020). The primers used in this study are listed in Supplementary Table 1.

### Swimming Motility

A swimming motility test was performed as previously reported (Liu et al., 2020). Briefly, overnight-cultured bacterial suspension (diluted to 1 × 10^8^ CFU⋅ml^–1^) was stab-inoculated into MM medium semi agar (0.3%) plates (MSA medium) supplemented with 20 mM L-glutamate. The diameter of the swimming halo was measured after 72 h of incubation at 30°C. The experiment was repeated twice, with ten plates for each strain per replicate.

### Statistical Analysis

The GraphPad Prism 8 software was used for statistical analysis. The data between two treatments were compared and statistically analyzed through the unpaired *t*-test. The data among multiple treatments were compared and statistically analyzed though oneway analysis of variance and Tukey’s HSD test.

## Results

### Growth of Original Strains in the Acidic Environment

**FIGURE 2:**
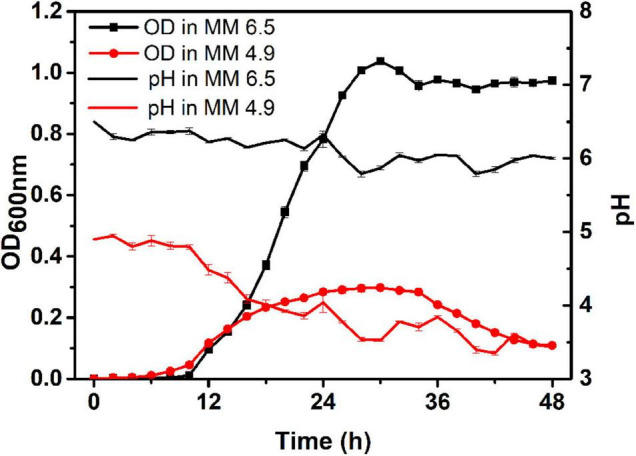
Growth curve and pH monitoring of CQPS-1 in MM medium with pH 6.5 or pH 4.9. MM 6.5 indicates the minimal medium with pH 6.5, MM 4.9 indicates the minimal medium with pH 4.9. Error bars indicate the standard error.

Minimal medium (MM medium) was used to simulate poor soil in this study. MM medium with pH 4.9 was selected as the acidic environment, and the MM medium with pH 6.5 was selected as the control environment. The growth curves of the original strain CQPS-1 in these two media and the dynamics of pH during the growth process were evaluated (as shown in Figure 2). When growing in the control environment (pH 6.5 MM medium), the strain entered the logarithmic phase after 10 h of growth, reached the maximum OD value (OD_600 nm_ = 1.04) at 30 h, then decreased slightly and remained stable; during growth in the control environment, the pH fluctuated at approximately 6.0, and at 28 h, the pH value was the lowest at 5.79. When growing in the acidic environment (pH 4.9 MM medium), the strain also began to grow rapidly after 10 h, reaching the maximum value at 30 h, but the maximum OD_600 nm_ value was only 0.298, and then the value began to decline from 36 to 48 h, the OD_600 nm_ at 48 h was only 0.109; approximately the pH during growth in the acidic environment, although the pH fluctuates slightly during the entire growth process, pH showed a downward trend as a whole (pH < 4.9), reaching the lowest point at 42 h (pH 3.35). The acidic environment is not conducive to the growth of *R. solanacearum* CQPS-1, and the pH value does not increase during the growth process, which can be used as a stress environment for the long-term experimental evolution of *R. solanacearum*.

### Serial Passage Experiment of *Ralstonia solanacearum* in an Acidic Environment

Long-term experimental evolution was performed in MM medium with pH 4.9 and pH 6.5 using the original strain CQPS-1, and the diagram of the experimental design is displayed in Figure 1. The strains grown in the acidic environment were denoted as C49, and the strains grown in the control environment were denoted as C65. Estimated by the plate count, *R. solanacearum* grew for approximately 6 generations when cultured in MM medium at pH 4.9 for 24 h and grew for approximately 7 generations when cultured in MM medium at pH 6.5 for 24 h. The time used for long-term serial culturing of 2000 generations was approximately 334 day in MM medium at pH 4.9, and 286 day in MM medium at pH 6.5.

After the long-term serial passage experiment, the morphologies of colonies changed on the BG plate containing TTC (shown in Supplementary Figure 1). All the colonies after the serial passage experiment were small, dark red, with poor fluidity, while the colonies of the original strain CQPS-1 were cream-colored, irregularly shaped, and highly fluid. The results indicated that after serial passage culturing in liquid MM medium, whether the pH was acidic or close to neutral, the bacterial colony morphology changed from the typical virulent type to the non-virulent type.

### Long-Term Experimental Evolution of *Ralstonia solanacearum* in an Acidic Environment

**FIGURE 3:**
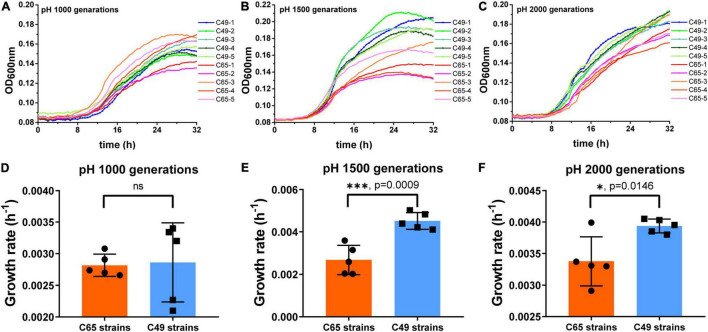
Growth curves and growth rates of 1000, 1500, and 2000 generations in MM medium (pH 4.9). Strains were cultured statically at 30°C in MM medium at pH 4.9. Bacterial growth was performed in 96-well plates and monitored using a Multiskan GO microplate reader (Thermo Fisher Scientific). The growth rates of each sample within 32 h were determined using the kinetic analysis module of Multiskan GO software. **(A–C)** Growth curves of 1000 generations, 1500 generations, and 2000 generations, respectively. **(D–F)** Growth rates of 1000 generations, 1500 generations, and 2000 generations. Asterisks indicate statistical significance (unpaired *t*-test).

The adaptation of the strains to the acidic environment was monitored by measuring the growth rate of the strain in MM medium at pH 4.9. As shown in Figure 3, when cultured for 1000 generations, the growth curves of some C49 strains (C49-3 and C49-5) showed a growth advantage compared to all C65 strains (Figure 3A). When growing to 1500 generations (Figure 3B), the overall growth curves of the C49 strains showed a better growth trend than the overall growth curves of the C65 strains. After 2000 generations of culture (Figure 3C), all C49 strains grew faster to the logarithmic phase and had higher growth than all C65 strains. Comparing the growth rates of C49 strains and C65 strains, 1000, 1500, and 2000 generations of C49 strains were 1.25-fold, 1.65-fold, and 1.22-fold larger than the growth rates of C65 strains, respectively, showing significant differences (Figures 3D–F).

### Selection of Acid-Evolved Clones

**FIGURE 4:**
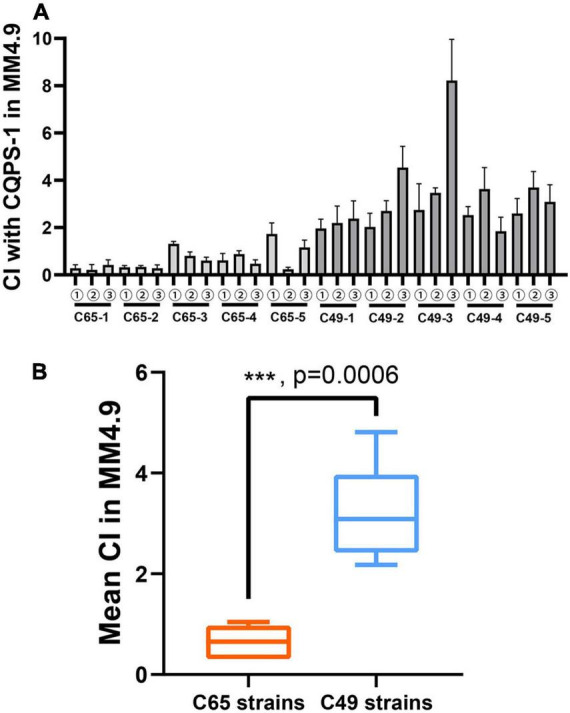
Competitive index (CI) values of experimental strains relative to the ancestral clone CQPS-1 growing in MM medium with pH 4.9 by distinguishing colony morphology. The CI was defined as the experimental/ancestral-clone ratio divided by the ratio in the corresponding inoculum. **(A)** Individual CI value of selected clones. ①-③ means three clones randomly isolated from each independent population generated by a long-term serial passage experiment from the control environment or the acidic environment. **(B)** Comparison of the mean CI of all selected clones from C49 strains and C65 strains. Asterisks indicate statistical significance (unpaired *t*-test).

Three clones were randomly isolated from each independent population generated by a long-term serial passage experiment from the control environment or the acidic environment to evaluate the fitness of the acidic environment. The acidic environment fitness of each selected clone was measured by co-culturing with the ancestral clone in MM medium at pH 4.9 using competition experiments by distinguishing colony morphology. A CI was used as a fitness estimator. The results are illustrated in Figure 4. The CIs of all selected clones from C49 strains were greater than 2 (Figure 4A). Therefore, the selected clones were superior to the original strain in acidic environment competitiveness. As shown in Figure 4B, the mean CI for the 2000 generations of C49 strains was 3.17, which was significantly higher than the mean CI for the 2000 generations of C65 strains (unpaired *t*-test analysis, *p* = 0.0006).

### Verification of Variant Gene Sequences of Experimental Strains

To determine the nature of genetic variations that occurred during the serial passage experiment of strain CQPS-1 *in vitro*, we sequenced the genomes of 10 clones based on the results of competition assays: 5 acid-evolved clones from C49 strains (C49-1③, C49-2③, C49-3③, C49-4②, C49-5②), and 5 control clones randomly selected from C65 strains (C65-1③, C65-2①, C65-3①, C65-4②, C65-5①). The details of the genome sequencing information for each sample are shown in Supplementary Table 2. The GC contents were 66.63–66.86%. The genome of the original strain CQPS-1 was used as the reference genome to analyze the variation of the experimental strains. More than 79 million paired-end reads (2 × 150 bp) were generated, leading to an approximately 300 × to 400 × total coverage of the reference genome (Supplementary Table 2). The comparison rates between the sample and the reference genome were 99.77–99.89%, and the genome coverage was 99.95%. Variations in the strains, including non-synonymous SNPs (single nucleotide polymorphisms) and small InDels (insertion-deletions), were counted (shown in Supplementary Table 3).

To screen for effective variations, the mutated gene that more than three genomes from five independent populations had mutations was selected. Then, the selected genes were verified by PCR amplification and Sanger sequencing in the C49 series and C65 series strains. Based on the sequencing and verification results, three genes were observed in parallel evolution: (1) gene ID BC350_10400 (transcriptional activator *phcA*); (2) BC350_10115 (response regulator transcription regulator protein *pehR*); and (3) BC350_11470, encoding a signal peptide protein of unknown function (shorthand “*spp*”). Table 1 lists all the genetic modifications identified in the sequenced clones compared with the CQPS-1 reference genome. The *phcA* gene of 5 clones from the long-term serial passage experiment of acidic environment mutated, although the mutation situation was different, and all the C65 series clones did not mutate. One IS (insertion sequence) movement was found in the *phcA* gene of C49-1, and 5 nucleotides were deleted in the *phcA* genes of C49-2, C49-3, and C49-4; moreover, 1 nucleotide was inserted in the *phcA* gene of C49-5. The *pehR* gene was mutated in both C49 series and C65 series clones, but the mutation sites were not the same (Table 1). In addition, 9 nucleotides were inserted in the *spp* gene of C49-1, C49-2, C49-3, and C49-4, and no mutation occurred in this gene of C49-5.

**TABLE 1:** Gene variation after verification.

Strains	BC350_10400 (*phcA*)	BC350_10115 (*pehR*)	BC350_11470 (*spp*)
C65-1	NM	NM	NM
C65-2	NM	Del 2 nt	NM
C65-3	NM	NM	NM
C65-4	NM	Del 2 nt	NM
C65-5	NM	Del 2 nt	NM
C49-1	IS	NM	Ins 9 nt
C49-2	Del 5 nt	Ins 2 nt	Ins 9 nt
C49-3	Del 5 nt	Ins 2 nt	Ins 9 nt
C49-4	Del 5 nt	Ins 2 nt	Ins 9 nt
C49-5	Ins 1 nt	Del 38 nt	NM

*NM means no mutation compared with original strain CQPS-1. IS, insertion sequence; Del, deletion; Ins, insert; nt, nucleotides.*

### Loss of the *phcA* Gene Increased the Growth of *Ralstonia solanacearum* in MM Medium With pH 4.9

Based on the results of genome analysis, we can infer that the mutations of the *phcA* gene or *spp* gene may be related to the strain adapting to the acidic environment. To verify this possibility, we generated deletion mutants in the original strain CQPS-1 by gene knockout experiments. After deleting the *spp* gene, the growth of the mutant strain in the acidic environment (MM medium with pH 4.9) was no different from the growth of the mutant strain of the wild-type strain CQPS-1 (Supplementary Figure 2).

**FIGURE 5:**
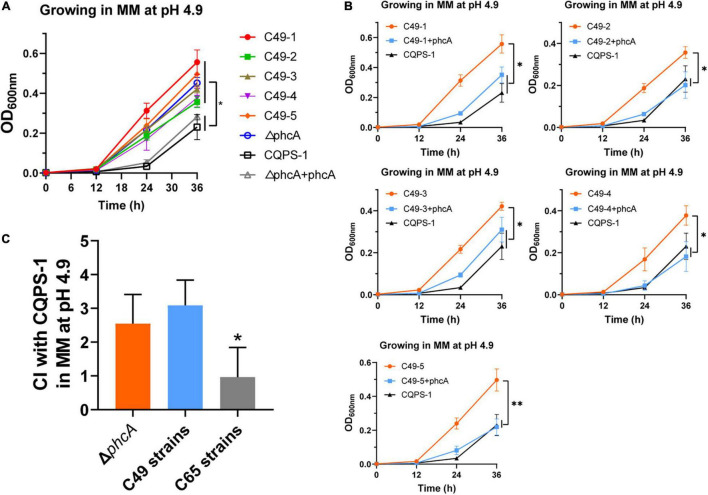
Growth curves and competition experiments of the *phcA* deletion mutant, C49 strains and the wild-type strain. **(A)** Growth curves of the *phcA* deletion mutant, C49 strains and the wild-type strain in MM medium at pH 4.9. **(B)** Growth curves of C49 strains after expressing an intact *phcA* gene by the complementary experiment. **(C)** The mean competitive index (CI) values of the *phcA* deletion mutant, acid-evolved clones, and control C65 clones relative to the wild-type CQPS-1 growing in MM medium at pH 4.9. The five selected clones of experimental C49 strains or C65 strains were used in the experiments. Asterisks indicate statistical significance (unpaired *t*-test, *p < 0.05 or ^**^p < 0.01).

The growth rates of the Δ*phcA* mutant, C49 strains and the complemented *phcA* strains in MM medium at pH 4.9 were also evaluated (Figures 5A,B). At the end of incubation, the growth rates of the Δ*phcA* mutant and C49 strains were significantly higher than that of wild-type strain CQPS-1 (Figure 5A). The Δ*phcA* mutant complemented the *phcA* gene (under the control of its native promoter) growed as slow as the wild-type strain did (Figure 5A). Significantly, complementation of the C49 strains by insertion of a copy of the wild-type *phcA* locus and its promoter into the selectively neutral *att* site restored the mucoid colony morphology (Supplementary Figure 3), and showed a growth trend consistent with wild-type strain in MM medium at pH 4.9 (Figure 5B).

The competition experiments was performed to evaluate the acidic environment fitness of Δ*phcA* mutant in MM medium at pH 4.9 by co-culturing with the wide-type strain CQPS-1. As shown in Figure 5C, the mean CI of Δ*phcA* mutant was 2.55, which showed no significant difference when compared with the five acid-evolved clones (mean CI was 3.09), but showed significantly higher than the mean CI of five control C65 clones (mean CI was 0.97). The results showed that loss of function of the *phcA* gene was associated with the fitness gain of *R. solanacearum* in an acidic environment.

### Swimming Motility of the Acid-Evolved Clones and the Δ*phcA* Mutant Enhanced

**FIGURE 6:**
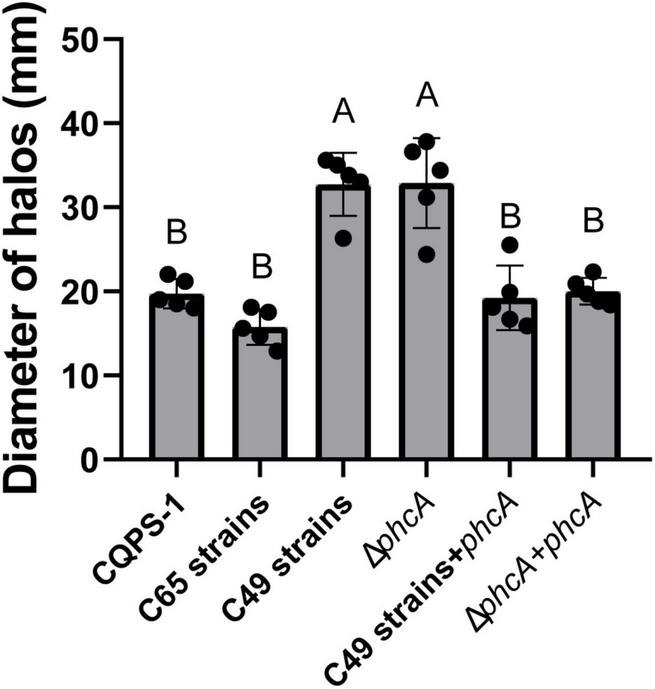
Swimming motility of the acid-evolved clones, the Δ*phcA* mutant, the control strains, and the original strain CQPS-1. Swimming motility assays were performed on MM medium semi agar (0.3%) plates (MSA medium) supplemented with 20 mM L-glutamate. Swimming halos observed after 72 h incubation at 30°C. The selected 5 “acid-evolved clones” and 5 “C65 control clones” were used as the C49 strains and C65 strains, respectively. Different letters indicated significant difference as determined by Tukey’s HSD (uppercase difference *p*-value < 0.01).

The swimming motility of the acid-evolved clones and the Δ*phcA* mutant was detected in MSA medium. The results 72 h after inoculation in MSA medium are shown in Figure 6. The mean swimming haloes produced by the acid-evolved clones (C49 strains), the control strains (the C65 strains), the Δ*phcA* mutant, and the original strain CQPS-1 were 32.74 ± 1.67 mm, 15.76 ± 0.94 mm, 32.88 ± 2.40 mm, and 19.74 ± 0.78 mm, respectively. Compared with the swimming haloes of CQPS-1, the haloes of the C49 strains were 1.66-fold increased, showing a significant difference (*p* < 0.01, ANOVA). There was no significant difference between swimming haloes of the C49 strains and the Δ*phcA* mutant. Complementation with the *phcA* gene (under the control of its native promoter) could completely restore the swimming motility of the Δ*phcA* mutant and C49 strains to that of wild-type strain CQPS-1 (Figure 6).

## Discussion

Experimental evolution is an important tool for studying natural processes and molecular functions (Kassen, 2019; Remigi et al., 2019). This method can supplement traditional molecular genetics and enhance the understanding of the molecular processes of microbial cells. Under laboratory conditions, it is possible to quickly evaluate the influence of a single factor on the evolution of the experimental population and explore its adaptive molecular mechanism by controlling a single variable (Blount et al., 2012; Lenski, 2017). In addition, there are some experimental evolution studies implicating the same adaptive loci as those observed in natural populations (Hsu et al., 2021). This research performed long-term experimental evolution to study the process of *R. solanacearum* adapting to an acidic environment in the laboratory. More than 2000 generations of experimental strains were obtained by serial passage experiments. Combined with whole genome sequencing, we identified the molecular determinants that drive adaptation in *R. solanacearum* when grown in an acidic environment.

Experimental evolution has been applied in *R. solanacearum* in recent years, mainly to explore the genetic mechanism of the adaptability of the strain to the host (Guidot et al., 2014; Marchetti et al., 2014). Guidot et al. (2014) performed a serial passage experiment of *R. solanacearum* on its original hosts (hosts that can cause bacterial wilt) and distant hosts (plants whose strains can grow asymptomatically) to study the genetic bases of host adaptation. The results of phenotypic analysis showed that pathogenic bacteria can increase their adaptability for both the original and distant hosts. This research is a successful case of studying the adaptability process and mechanism of microorganisms to a controlled environment under laboratory conditions. Moreover, based on the results of genome resequencing, the function of *efpR* in plant adaptability was found (Guidot et al., 2014; Perrier et al., 2016), which means that the combination of experimental evolution and genome resequencing is a powerful method that can effectively provide a comprehensive understanding of the adaptation process of microbial genome changes in the experimental evolution of microorganisms. This study simulated poor soil with MM medium, and directly explored the relationship between the long-term effects of acid stress and *R. solanacearum* by experimental evolution. When the strains grew to more than 1000 generations, the acid experimental strains (C49 strains) showed significantly improved acid adaptability. The *R. solanacearum* strain was demonstrated to have highly variable plasticity and shows strong adaptability in adverse environments.

In soil, stationary broth culture, or long-term culture on agar plates, *R. solanacearum* spontaneously undergoes phenotypic conversion to the PC type, that is, changes from a fluid colony to a non-fluid colony (Kelman, 1954; Brumbley and Denny, 1990). The PC type is weakly pathogenic or non-pathogenic, but it can still colonize the host tissue without causing symptoms. Earlier reports pointed out that *R. solanacearum* has been cultured under laboratory conditions for a long period until it reaches a stable stage. After 20 days, the strain will undergo phenotypic conversion, and mucus-secreting and non-mucus-secreting strains coexist in the population (Zhu et al., 2010). Our results also show that under long-term experimental conditions, all experimental strains will transform into PC types. According to previous reports, spontaneous inactivation of *phcA* results in the phenotypic conversion of *R. solanacearum* (Brumbley et al., 1993; Jeong and Timmis, 2000). Combined with the results of this study, there are two situations in the spontaneous phenotypic conversion of *R. solanacearum*. One situation is that when the phenotype converts, the *phcA* gene is mutated. In this situation, mutations were IS movements (strain C49-1) or deletions (strains C49-2, C49-3, C49-4, C49-5), which resulted in modified coding sequences (CDS). The other situation is that there is no conversion in the *phcA* gene when the phenotype converts, such as strains in the C65 series (Table 1). However, whether the phenotypic conversion of the C65 series strains is due to gene regulation needs to be further verified.

PhcA, a LysR-type transcriptional regulator, plays an important role in the quorum sensing (QS) of *R. solanacearum* (Genin and Denny, 2012). QS regulates the growth patterns of *R. solanacearum* at different population densities. It has been reported that cells have little functional PhcA when the population is at low density, whereas cells at higher density have abundant functional PhcA (Genin and Denny, 2012). *R. solanacearum* can use many sources (carbon, nitrogen, sulfur, phosphorus and iron) more efficiently when it is at low cell density than in a high-cell-density mode (Khokhani et al., 2017). As previously reported, glucosidases, which convert complex sugars such as glycosides, glycans, and glycoconjugates to glucose, have more expression in Δ*phcA* cells than in wild-type cells (Khokhani et al., 2017). Glucose is an important carbon source for *R. solanacearum*. Rapid uptake of glucose may help *R. solanacearum* gain an advantage in competition. The “acid-evolved strains” with spontaneous mutation of PhcA have enhanced metabolic capacity and can rapidly utilize nutrients for its own growth in a barren acidic environment. Our study confirmes that the inactivation of the *phcA* gene is the reason why *R. solanacearum* can adapt to the long-term acidic environment (Figure 5).

The swimming motility of the acid-evolved strains was significantly enhanced compared with the swimming motility of the control strains and the original strain CQPS-1 (Figure 6). Swimming motility, one of the most important characteristics of *R. solanacearum*, allows *R. solanacearum* to effectively invade and colonize the host plant (Liu et al., 2001; Tans-Kersten et al., 2001, 2004; Dalsing and Allen, 2014). *R. solanacearum* has been reported to attach to the roots of the host faster after knocking out *phcA* (Khokhani et al., 2017). We could speculate that the “evolved-strains” of *R. solanacearum* would attach to the roots better due to the enhanced swimming motility when there is a host.

PhcA mediates a trade-off from maximizing growth to producing costly virulence factors (Peyraud et al., 2016; Khokhani et al., 2017; Lowe-Power et al., 2018). Although it is speculated that “acid-evolved strains” can achieve rapid colonization of hosts through better utilization of nutrients and superior motility, spontaneous mutation of PhcA would result in losses of pathogenicity, indicating that the strain may not cause host death. Interestingly, previous studies confirmed that the non-pathogenic form of *R. solanacearum* caused by mutations in the *phcA* gene could reverse the pathogenic form *in planta* or in the presence of root exudate of host plants (Poussier et al., 2003; Mori et al., 2012). Further speculation, after preferentially reaching the roots of the host, the “evolved-strains” may cause disease by phenotypic conversion after interacting with the host. This hypothesis needs further verification.

The *spp* gene deletion mutant of wild-type strain has been constructed, and the growth of the mutant in the acidic environment are not affected after the *spp* gene deletion (Supplementary Figure 2), indicating that the *spp* gene from wild-type strain has no direct relationship with the acidic adaptation. However, three codons have been inserted into this gene in the “acid-evolved strains” (Table 1), which means the protein could be still expressed. How the new *spp* protein functions after mutation still needs to be further explored.

Taken together, this work provided evidence for understanding the evolutionary dynamics of *R. solanacearum* in acidic environments. In short, *R. solanacearum* expands its population in an acidic environment by loss of function in the *phcA* gene. The abilities of acid-evolved clones to acquire after adapting to acidic environments remain to be further evaluated.

## Data Availability Statement

The data of genome resequencing presented in the study are deposited in the Sequence Read Archive (SRA) of NCBI, accession number: PRJNA774485.

## Author Contributions

YL and WD designed the research and drafted the manuscript. YL and XT conducted experiments and performed data analysis. YP, YD, and XL completed mutant construction, complementation analysis, and evaluation. JY and XL contributed to revising the manuscript. All authors made fundamental contributions to the manuscript, contributed to the interpretation of data, and approved the final manuscript.

## Conflict of Interest

JY is employed by Sichuan Branch of China Tobacco Corporation. The remaining authors declare that the research was conducted in the absence of any commercial or financial relationships that could be construed as a potential conflict of interest.

## Publisher’s Note

All claims expressed in this article are solely those of the authors and do not necessarily represent those of their affiliated organizations, or those of the publisher, the editors and the reviewers. Any product that may be evaluated in this article, or claim that may be made by its manufacturer, is not guaranteed or endorsed by the publisher.

## Footnotes

1
http://sourceforge.net/projects/picard/
