# Supplementary material for: Mutation in phcA Enhanced the Adaptation of Ralstonia solanacearum to Long-Term Acid Stress
Source: Front Microbiol. 2022 May 26;13:829719. doi: 10.3389/fmicb.2022.829719 (PMC9204249; doi:10.3389/fmicb.2022.829719)
Supplement: Supplementary file 1 [file Data_Sheet_1.docx]

Supplementary Material

# Supplementary Figures and Tables

## Supplementary Figures


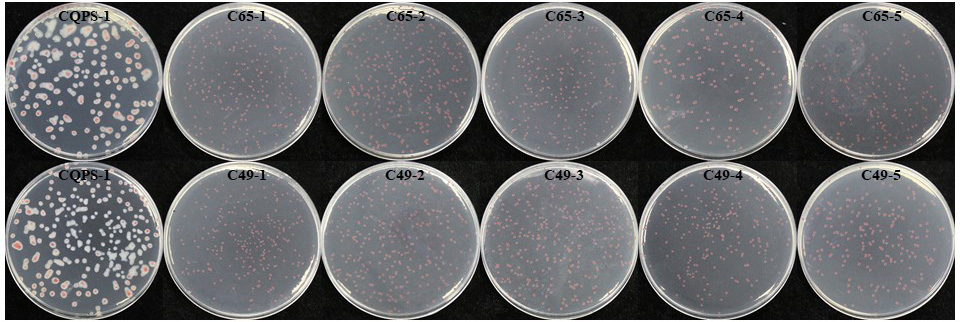


**Supplementary Figure 1.** Colony morphology of original and experimental *Ralstonia solanacearum* strains after the long-term serial passage experiment.





**Supplementary Figure 2.** Growth curves of the *spp* deletion mutant and the wild-type strain in minimal medium (MM medium) at pH 4.9. Error bars indicated the standard error of three independent biological replicates.


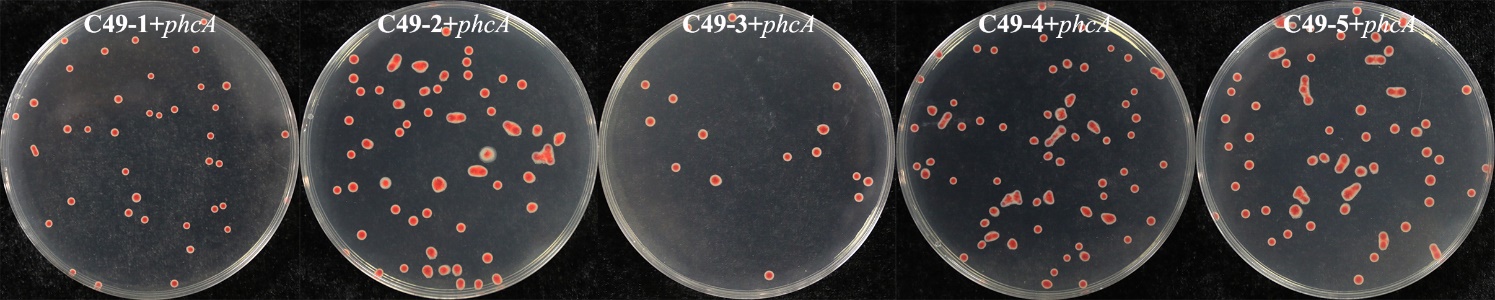


**Supplementary Figure 3.** Colony morphology of C49 strains complemented the *phcA* gene (under the control of its native promoter) by the complementary experiment.

## Supplementary Tables

**Supplementary Table 1.** Primers used in this study.

| Primers | Sequences (5’-3’) |
| --- | --- |
| spp-YZ-F | CGCACAATCCGCAACTCTTA |
| spp-YZ-R | GCACGGTACCCAAACCAGAT |
| phcA-YZ-F | ATGGTCAACGTCGATACCAAGC |
| phcA-YZ-R | AGACGGACAGCCGCGACT |
| pehR-YZ1-F | CATGTCCAAAGCTGCCGTC |
| pehR-YZ1-R | CGTTGAGCCGGTAGAACAAA |
| pehR-YZ2-F | TTTGTTCTACCGGCTCAACG |
| pehR-YZ2-R | GGCAAAGAAATCCAGCACGT |
| trmD-YZ-F | ATGTCATCTCGCTGTTCCCG |
| trmD-YZ-R | CGCTTGCGCATCGTATTCAA |
| phcA-A1SmaI | TCC**CCCGGG**TGGGCTACACGGAAGACAAG |
| phcA-B1C | GGAGGAGCGCGGTGCTGGCAGGGACGCTTTGGCTCAGACA |
| phcA-A2C | TGTCTGAGCCAAAGCGTCCCTGCCAGCACCGCGCTCCTCC |
| phcA-B2HindIII | CCC**AAGCTT**GCCGCCAAAACCCTTATGATTG |
| spp-EcoRI-F1 | G**GAATTC**CGCACAATCCGCAACTCTTA |
| spp-F2 | GTCCGGCCGGATCGCAACGGTAAATCCTTTTATAGATCAGTGA |
| spp-F3 | TCACTGATCTATAAAAGGATTTACCGTTGCGATCCGGCCGGAC |
| spp-HindIII-F5 | CCC**AAGCTT**GAACTGTCGGTCGGCGAG |
| phcAB2H | CTGGATCCAGACCTCAAGAACATCGGTC |
| phcAA1B | CTAAGCTTGCCAGCTACGACGAGATCTG |

**Supplementary Table 2.** Genome sequencing information of each sample.

| Strains | Clean Reads number | GC (%)^a^ | Q20 (%)^b^ | Q30 (%)^c^ | Average coverage depth | Mapped (%)^d^ | Properly_mapped (%)^e^ |
| --- | --- | --- | --- | --- | --- | --- | --- |
| C49-1 | 7506476 | 66.7 | 97.92 | 94.24 | 373 | 99.82 | 99.53 |
| C49-2 | 8202147 | 66.73 | 98.16 | 94.8 | 396 | 99.83 | 99.59 |
| C49-3 | 7660669 | 66.72 | 98.23 | 94.92 | 379 | 99.89 | 99.67 |
| C49-4 | 7989473 | 66.63 | 97.78 | 93.91 | 389 | 99.84 | 99.56 |
| C49-5 | 6512013 | 66.63 | 97.58 | 93.55 | 324 | 99.78 | 99.48 |
| C65-1 | 7342215 | 66.86 | 97.66 | 93.65 | 368 | 99.81 | 99.51 |
| C65-2 | 8769798 | 66.68 | 97.65 | 93.68 | 434 | 99.77 | 99.46 |
| C65-3 | 7979882 | 66.69 | 97.96 | 94.33 | 399 | 99.81 | 99.53 |
| C65-4 | 10182220 | 66.72 | 98 | 94.41 | 491 | 99.86 | 99.51 |
| C65-5 | 7784499 | 66.7 | 98.19 | 94.72 | 380 | 99.87 | 99.56 |

Notes:

^a^GC (%): Sample GC content

^b^Q20 (%): The percentage of bases with a quality value greater than or equal to 20 to the total number of bases

^c^Q30 (%): The percentage of bases with a quality value greater than or equal to 30 to the total number of bases

^d^Mapped (%): The percentage of the number of clean reads located to the reference genome to the number of all clean reads

^e^Properly_mapped (%): The paired-end sequencing reads are all located on the reference genome, and the distance is consistent with the length distribution of the sequenced fragments

**Supplementary Table 3.** Number of variant genes in 2000 generation acid experimental strains.

| strains | Gene number of non synonymous SNP | Gene number of InDel | strains | Gene number of non synonymous SNP | Gene number of InDel |
| --- | --- | --- | --- | --- | --- |
| C65-1 | 1 | 127 | C49-1 | 1 | 128 |
| C65-2 | 2 | 127 | C49-2 | 0 | 130 |
| C65-3 | 3 | 126 | C49-3 | 1 | 129 |
| C65-4 | 2 | 128 | C49-4 | 1 | 130 |
| C65-5 | 2 | 127 | C49-5 | 2 | 128 |
